# From scientific discovery to treatments for rare diseases – the view from the National Center for Advancing Translational Sciences – Office of Rare Diseases Research

**DOI:** 10.1186/s13023-018-0936-x

**Published:** 2018-11-06

**Authors:** Petra Kaufmann, Anne R. Pariser, Christopher Austin

**Affiliations:** 1 Avexis Inc. Formerly of Office of Rare Diseases Research (ORDR), National Center for Advancing Translational Sciences (NCATS), National Institutes of Health (NIH), Bethesda, MD USA; 2 ORDR, NCATS, NIH, Bethesda, MD USA; 3 NCATS, NIH, Bethesda, MD USA

**Keywords:** Rare diseases, Clinical studies, National Center for Advancing Translational Sciences, Investigational therapies, Interdisciplinary research

## Abstract

We now live in a time of unprecedented opportunities to turn scientific discoveries into better treatments for the estimated 30 million people in the US living with rare diseases. Despite these scientific advances, more than 90% of rare diseases still lack an effective treatment. New data and genetics technologies have resulted in the first transformational new treatments for a handful of rare diseases. This challenges us as a society to accelerate progress so that no disease and no patient is, ultimately, left behind in getting access to safe and effective therapeutics. This article reviews initiatives of the National Center for Advancing Translational Sciences (NCATS) Office of Rare Diseases Research (ORDR) that are aimed at catalyzing rare diseases research. These initiatives fall into two groups: Promoting information sharing; and building multi-disciplinary multi-stakeholder collaborations. Among ORDR’s information sharing initiatives are GARD (The Genetics and Rare Diseases Information Center), RaDaR (The Rare Diseases Registries Program) and the NCATS Toolkit for Patient-Focused Therapy Development (Toolkit). Among the collaboration initiatives are the RDCRN (Rare Diseases Clinical Research Network), and the NCATS ORDR support for conferences and workshops. Despite the success of these programs, there remains substantial work to be done to build enhanced collaborations, clinical harmonization and interoperability, and stakeholder engagement so that the recent scientific advances can benefit all patients on the long list of rare diseases waiting for help.

## Background

The scientific landscape for rare diseases has been changing rapidly, and this change is expected to accelerate. People living with rare diseases are increasingly benefiting from new therapeutics, some resulting from the break-through technologies now emerging in medicine. However, less than 5% of the more than 7,000 rare diseases believed to affect humans currently have an effective treatment. While individually rare, in the aggregate there are an estimated 30 million people in the United States (US) living with rare diseases, or 1 in 10 Americans. Most rare diseases affect children, and many are lethal or severely disabling. This great unmet need makes it imperative that we find ways to accelerate the therapy development process so that we can help the many patients and families who are in search of better treatments.

**Fig. 1:**
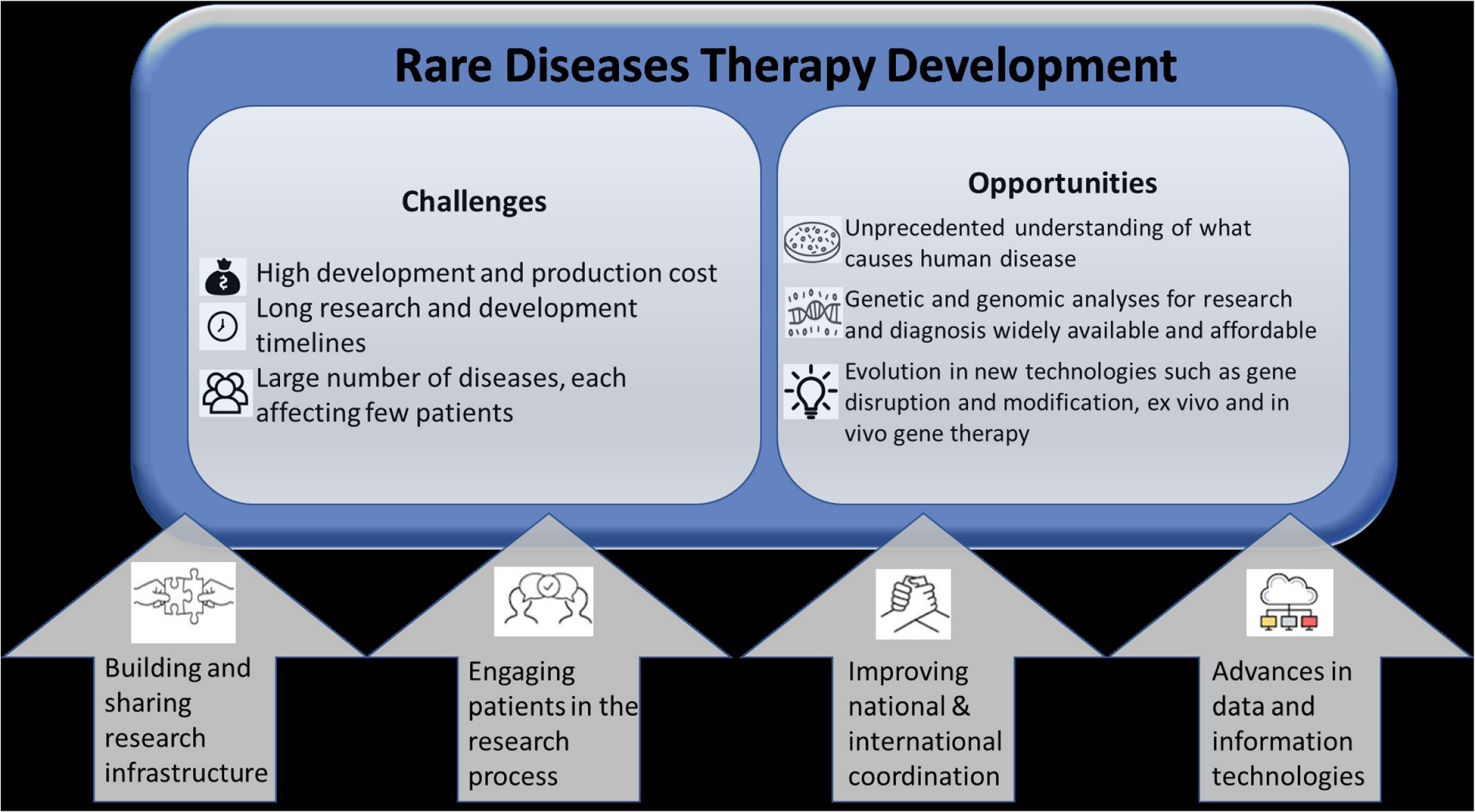
Challenges, opportunities and strategies for rare diseases therapy development

To examine the question of how to best accelerate rare diseases research, we will review some of the challenges in rare diseases research and opportunities for therapy development that recent scientific advances are presenting. Fig. 1 represents a schematic summary of these challenges and opportunities. We will also discuss relevant programs and initiatives in the Office of Rare Diseases Research (ORDR) at the National Center for Advancing Translational Sciences (NCATS), part of the National Institutes of Health (NIH) that are intended to facilitate and accelerate moving more treatments to more rare disease patients faster.

## Challenges for rare diseases therapy development

Bringing a new drug or biological product (“drug”) to market can take over a decade and cost more than $2.5B, and these numbers are increasing [1]. High development costs are particularly difficult for rare diseases to overcome because the market for the product, once approved, is inherently small, thus limiting the potential for return-on-investment. Adding to this are often high manufacturing costs for innovative technologies, such as gene therapy approaches, until better production and characterization methods can be developed and automated. For mutation-specific nucleic acid therapies such as oligos, morpholinos, and aptamers there are additional challenges in reaching all rare diseases patients given the mutational heterogeneity in most conditions. Patient-specific cell therapies have the further challenge that a separate batch needs to be produced for each patient.

Long development times are also of particular concern for rare diseases where often a knowledge base for the disease, such as natural history data, needs to be built, outcome measures need to be identified and developed, and where trial recruitment of the relatively small number of available trial participants can be prolonged. Such delays add costs, and, more importantly, cost lives. In life-threatening rare diseases, most of which are chronic progressive disorders, patient access to an effective new treatment can change the trajectory of the disease and reduce morbidity and mortality.

Weighing the cost, time and risk associated with therapy development, academic and industry researchers and developers have been largely focused on a subset of less than 100 rare diseases, often competing for trial participants and researchers’ time while “racing” to be first to achieve marketing authorizations. How can we promote both national and international interest in the many other rare diseases that are currently perceived as too uncommon to merit public sector investment, or too ‘risky’ to attract much private sector attention? How can we find vital scalable and sustainable pathways to help the many families afflicted with rare diseases who are searching for effective treatments?

The recent success stories in getting regulatory approvals for transformative therapies have also raised new dilemmas in ensuring access to all patients in need. New payment frameworks are being explored, and often aim to take into account the disease and financial burden now borne by individuals and societies over a patient’s lifetime relative to the cost of the successful treatment. To be sustainable, rare disease treatments ideally would not place undue stress on health systems, payers, and patients, and would eventually become accessible to patients in resource-poor environments. Ideally, such reimbursement frameworks would also foster the continued growth of rare diseases therapy development.

## Opportunities and recent successes

Our understanding of what causes human disease is unprecedented. We can diagnose more and more diseases using genomic analysis. There is growing experience in developing small molecule therapies. Also, we can increasingly design tailored treatment approaches that address the underlying genetic cause of many diseases. Gene transfer therapies are now successful in patients, replacing missing genes by using viral vectors [2–5]. Gene disruption technologies such as antisense oligonucleotides, RNA interference (RNAi), or microRNA modulation are used to modify or block a disease-causing protein [6, 7]. Gene-modified cell therapy has been successful at using cells such as chimeric antigen receptor (CAR) T cells that have been changed to treat cancer [8, 9]. Gene editing approaches can be deployed to directly modify genes in vivo and ex vivo using clustered regularly interspaced short palindromic repeats (CRISPR) and zinc finger (ZFN) technologies [10, 11]. A growing number of patients are now participating in clinical trials that test gene therapy modalities. In the past year, the first patient has been enrolled in a clinical trial using in vivo gene editing. Also, this decade has seen a steady increase in drug approvals for rare diseases, including rare cancers.

The task is now to find ways to reduce the time, costs, and risks associated with rare disease therapy development; only then will effective treatments be more likely reach all patients in need.

## Current strategies to advance rare diseases therapy development

The authors believe that decreasing the cost, time and risk of developing new rare diseases therapies will require a focus on commonalities, scale, and technological as well as sociolocultural innovation, including:

1. Building and sharing research infrastructures
2. Actively engaging patients in the research process
3. Improving coordination among national and international stakeholders
4. Applying advances in data and information technologies to rare disease research.

**Fig. 2:**
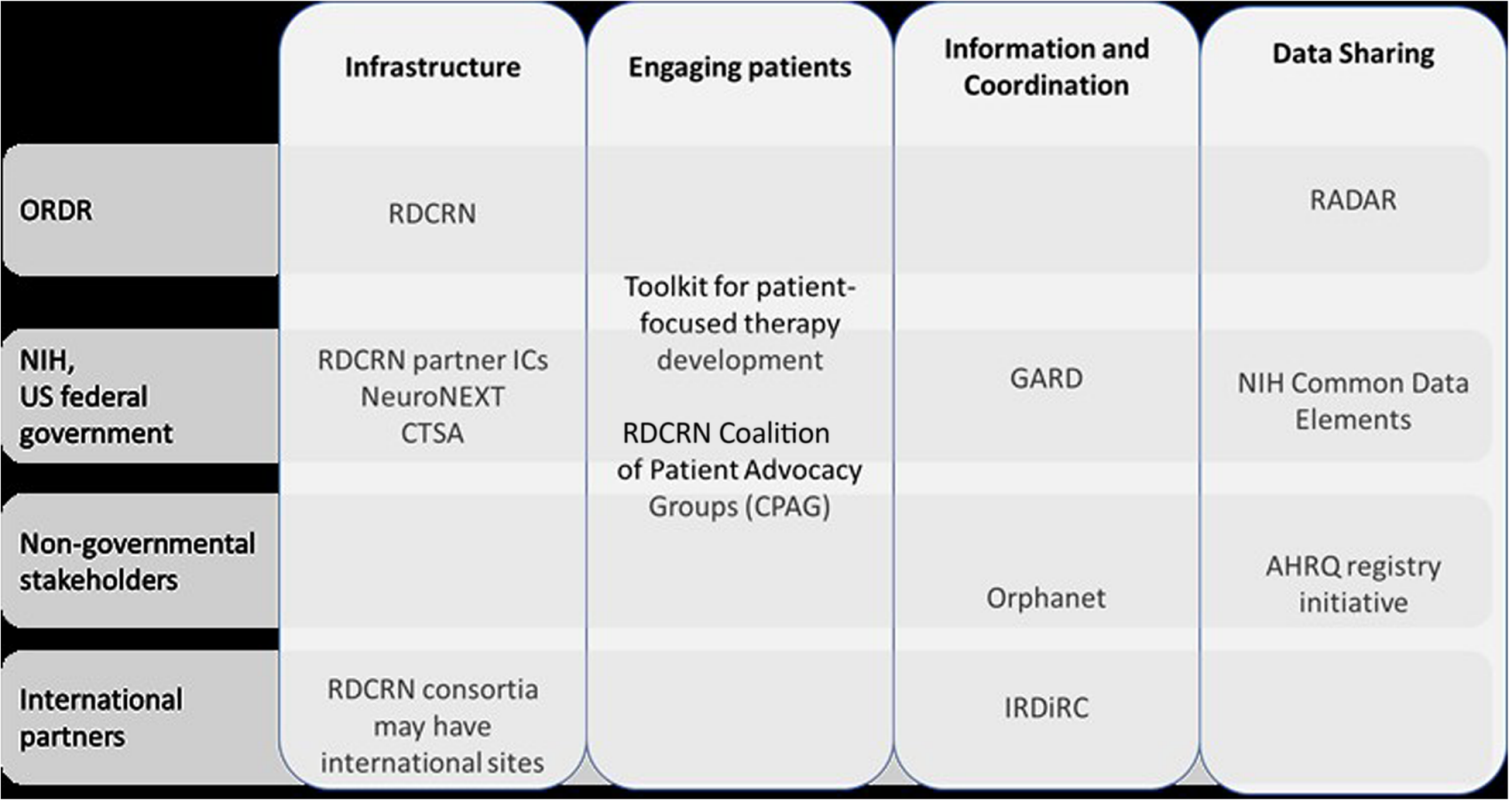
Overview of ORDR initiatives by strategy, and examples of national and international partners

Figure 2 is a schematic overview of ORDR initatives aimed at these four strategies listed above, along with examples of US and international partnerships.

### Building and sharing research infrastructures

By definition, rare diseases are uncommon so that there are fewer patients, clinicians, and researchers. It is important to keep in mind that research in rare disease etiology, mechanisms and treatment approaches can advance progress in common diseases. The infrastructure needed for clinical studies is too often established de novo for each disease and clinical development program, making it a costly enterprise, especially when the expense is considered per research participant. One approach is therefore the use of national and international networks for research that can take on therapy development.

#### The Rare Diseases Clinical Research Network (RDCRN)

The NIH’s NCATS, together with several other NIH Institutes and Centers (IC) is supporting the Rare Diseases Clinical Research Network (RDCRN) to facilitate rare disease study coordination, enrollment of research participants, and data sharing. The RDCRN consists of several distinct clinical research consortia with a shared Data Management and Coordinating Center, through which research into more than 200 diseases is being conducted at sites across the nation and, when needed, internationally. To maximize the impact of the RDCRN, each consortium is focused on more than one related rare disease and fosters a broad range of clinical research including registries and natural history studies. The RDCRN encourages the collaboration of physician scientists, their multidisciplinary teams and patient groups, and also provides training in rare diseases research to new investigators [12].

#### The NeuroNEXT initiative and the Clinical and Translational Science Awards program (CTSA)

Rare diseases research also may benefit from advances in the NCATS Clinical and Translational Science Awards (CTSA) Program that supports academic medical centers across the nation. After a review of the Program by an Institute of Medicine panel, [13] NCATS has placed greater emphasis on collaboration and launched a Trial Innovation Network so that needed contracting, ethics reviews, training, and other building blocks can be shared more readily. To streamline in particular the ethics reviews conducted by multiple Institutional Review Boards (IRBs) at each institution participating in a multi-site study, NCATS supports the SMART IRB Platform with now over 350 institutions having agreed to one IRB authorization agreement [14]. Based on previous NIH experiences, e.g. in the NeuroNEXT network, the ethics review of multi-site research by a single IRB can reduce start-up times and thus save time and cost [15, 16]. For rare diseases research, where national and international networks are often needed, it will be critical to gradually streamline the reviews and contracts needed for multi-site studies so that we can bring new treatments to patients faster.

### Actively engaging patients and communities in rare diseases research

The concept that those who are meant to benefit from research --patients, families and communities-- should be active participants in these efforts, is increasingly being recognized. The RDCRN requires that at least one patient group be involved in each of the consortia. This allows researchers to seek input on their work from patients, caregivers, health care providers, community members and other non-researcher stakeholders. The feedback covers issues pertaining to study design, endpoints, implementation, recruitment, and retention. The goals are to ensure that the questions asked and the disease manifestations targeted are important to patients, that the burden of study participation is not prohibitive, and that the outcome measures are relevant. Also, partnering with the patient community can help in the development of effective recruitment strategies, and can help uncover potential barriers to successful study implementation. This is a particularly powerful model for rare disease research where it is critical to reach potential research participants, to find out what a meaningful effect is and what patient perceptions are with regards to risk and benefit [17].

#### The Toolkit for Patient-focusedTherapy Development

**Fig. 3:**
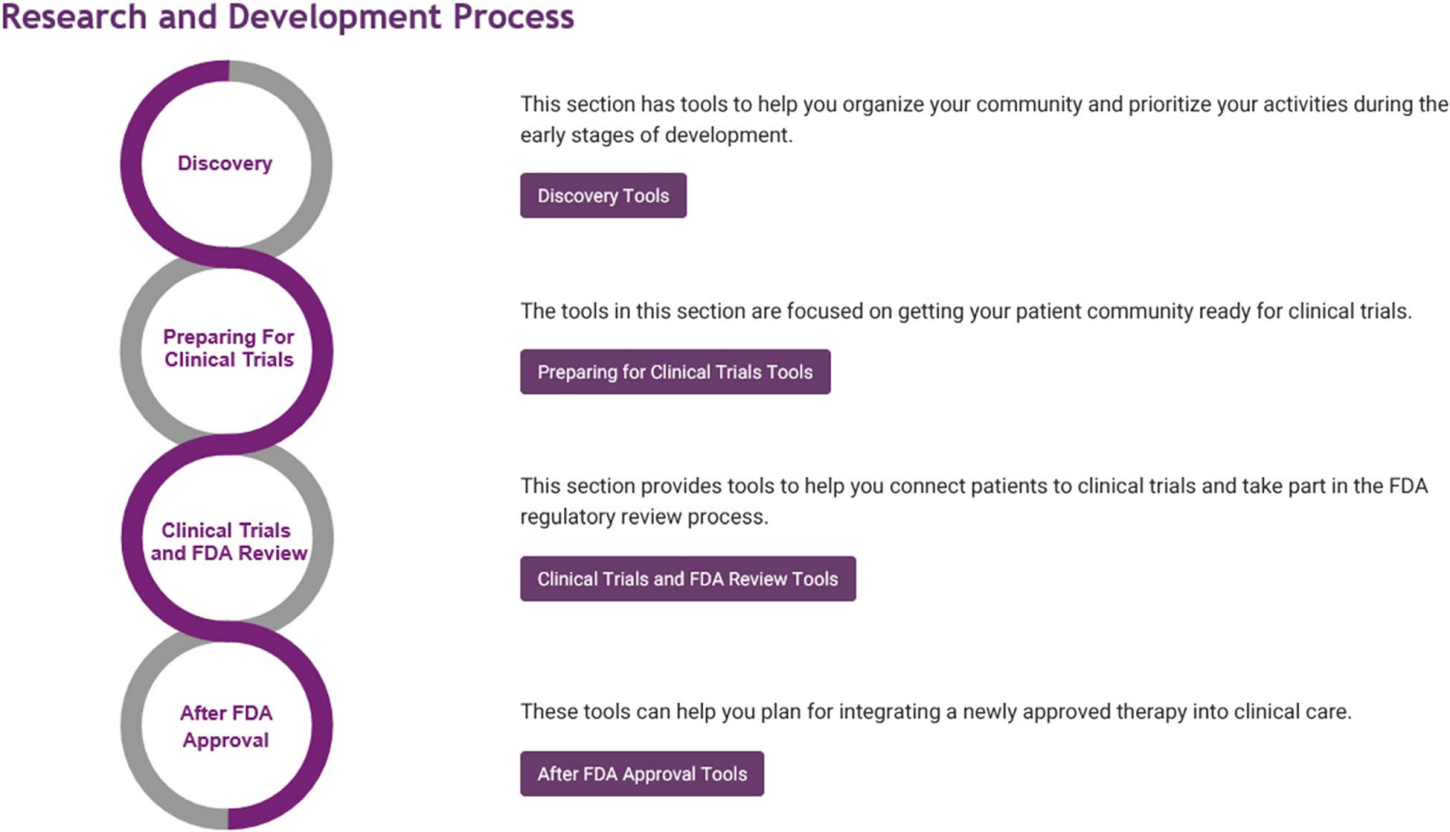
Section overview of the “Toolkit for Patient-Focused Therapy Development” [18]

To facilitate the active participation of patient groups in the research and development of rare disease therapies, the NCATS ORDR has developed a “Toolkit for Patient-Focused Therapy Development” (Toolkit) [18]. It was created to provide user-friendly access to a collection of online source materials that can help patient groups and others find the tools they need to promote and advance the development of treatments for their particular diseases. This initiative does not develop new tools, but rather facilitates the dissemination of existing tools. The process, however, also uncovered gaps in tools and resources that can inform future research strategies. The initiative is led by patient group representatives, and has obtained input from a wide range of stakeholders including patient groups, researchers, NIH and FDA staff. Launched in September 2017, the Toolkit uses a centralized portal that guides users in how to establish a registry, drive rare disease research, work more effectively with the NIH and the US Food and Drug Administration (FDA), as well as support post-market surveillance of approved therapies [19]. Figure 3 illustrates the organization of the toolkit portal.

### Improving coordination among national and international stakeholders

Coordination and collaboration are critical in speeding up the development of new treatments for rare diseases so that resources and capacity can be more effectively leveraged. When studies focusing on the same disease are coordinated rather than entirely independent or even duplicative, then research can progress faster and better treatments can become a reality for patients sooner.

The ORDR promotes such coordination and aims to serve as a node for rare diseases information and activities at the NIH, within the US Federal government, for stakeholders across the nation, and internationally.

#### NIH coordination and collaboration

At NIH, the ORDR staff help coordinate rare diseases research working with other NIH Institutes and Centers that each focus on different medical research disciplines, for example on heart or brain diseases. Because most rare diseases affect more than one organ system, they typically are within the scope and mission of more than one NIH Institute or Center. The ORDR addresses this by regularly convening trans-NIH rare diseases groups, and by coordinating rare diseases initiatives such as the RDCRN. This allows for bi-directional exchange of issues common to rare diseases research, and for the dissemination of rare diseases research policies and relevant national and international activities.

#### US Federal government coordination and collaboration

Within the US Federal government, ORDR staff have regular meetings with relevant FDA staff to coordinate efforts and promote the exchange of knowledge between the part of the Federal government that regulates medical products, the FDA, and the part of the Federal government that funds and conducts rare diseases research, the NIH. The ORDR staff have also reached out to other relevant government partners such as the Agency for Healthcare Research and Quality (AHRQ). For its Rare Diseases Registry (RaDaR) Program, the ORDR is collaborating with AHRQ to adapt for rare diseases research one of their established initiatives, the “Registries for Evaluating Patient Outcomes: A User’s Guide”, a resource with practical information on the design, operation, and analysis of patient registries [19].

#### Coordination and collaboration with national stakeholders

The ORDR staff participates in relevant conferences and meetings of rare diseases organizations, researchers, and patient groups. When possible, the ORDR contributes funding towards rare diseases conferences which are often important catalysts in advancing research in a given rare disease area. The ORDR also provides comprehensive information about rare diseases to the public through its online Genetic and Rare Diseases Information Center (GARD). This database provides accurate and current information about clinical and research aspects of rare diseases including data from the National Library of Medicine and links to support groups. GARD is a collaboration with the National Human Genome Research Institute (NHGRI) and Orphanet. In addition to online information in English and Spanish, GARD also offers email or phone discussions with information specialists [20]. Another US initiative advancing clinical research and stakeholder engagement is the Patient-centered Outcomes Research Institute (PCORI). The implementation of research findings and knowledge transition is a particular focus of this initiative, and PCORI has highlighted rare diseases as a high-priority research topic [21].

#### Coordination and collaboration with international stakeholders

For many rare diseases, there are too few patients and researchers in the US alone to make sufficient research progress. It is therefore critical to establish impactful collaborations with international stakeholders. The International Rare Diseases Research Consortium (IRDiRC) works to coordinate the rare disease efforts of funders, companies, patient groups, and scientists in over 20 countries on five continents. IRDiRC has recently enunciated ambitious global goals for rare disease diagnosis, treatment, and access in the next decade, which it believes international cooperation will make possible [22]. To reach its goals, IRDiRC provides opportunities for research funders to coordinate their investments, promotes harmonization of tools and standards, undertakes joint efforts to address common roadblocks to progress, and ensures relevance by universal patient involvement. Leadership at ORDR, NCATS, and other NIH Institutes and Centers are actively involved in IRDiRC activities, which both accelerates global rare disease goals, and informs NIH- and NCATS-specific strategies.

### Applying advances in data and information technologies for rare diseases research

Currently, our knowledge of rare diseases is typically fragmented because it is based on many small datasets that cannot be easily connected. Making these datasets interoperable for national and international collaborations could greatly speed up progress towards a treatment for more rare diseases. Larger datasets and more comprehensive data collections are more powerful in generating new medical knowledge and in planning effective trials to evaluate new treatments.

#### NIH common data elements

The ORDR is therefore advancing the use of ***standards*** so that data can be more rapidly shared in the future. In its RDCRN initiatives, the ORDR and its NIH partners are encouraging researchers to build on existing data standards including the “common data elements (CDEs)” developed with NIH support which are available through a portal at the National Library of Medicine [23]. For neurological disorders, for example, CDEs are available for a wide range of rare diseases. Developed by the research community, they are freely accessible online in user-friendly formats that allow for the development of case report forms and databases. Organized in a modular way, they support tailoring the data collection to each study’s specific needs [24].

#### International standards and data sharing

Ideally, rare diseases research data would also be connected through widely used standards so that they can link to existing clinical data in health care systems (e.g., HL7) and standards that link to research data, for instance, Human Phenotype Ontology (HPO) [25, 26]. Through its RADAR registry program, the ORDR aims to promote the adoption of data standards so that patient groups can more easily establish impactful registries. Through its RDCRN program, the ORDR not only encourages the use of standards upstream in study planning, but also supports downstream the sharing of de-identified datasets generated by RDCRN researchers. This is particularly important for rare diseases where every observation and data point must count, recognizing that clinical research may be delayed because datasets often do not materialize their full value beyond a narrowly focused primary analysis.

## Additional strategies to enhance rare diseases therapy development

Above, we have described initiatives by the NCATS ORDR, its partners and other stakeholders intended to accelerate therapeutics development for rare diseases. We also note some additional paths to explore:

1. Integration of research and care
2. Enhancing data collection and utilization of novel technology
3. Platform and collective research approaches
4. Leveraging large “precision” medicine and genotyping/phenotyping initiatives
5. Public sector-private sector parternships and leverage

### Integration of research and patient care

There are a limited number of disease experts for individual rare diseases, and many rare diseases patients come in contact with specialized clinics and physician scientists that provide both expert care and work on research projects. Given that electronic health records (EHR) and other data archiving (e.g., imaging) are almost universally used at academic medical centers, there are opportunities to better integrate the costly capture of clinical research data with the data that are already being collected for clinical purposes. This may be particularly relevant to longitudinal observational studies, such as natural history studies, where clinical information may be extracted or integrated into long-term data collections. Natural language processing and other machine-read and extraction techniques show great promise in this area.

### Improving rare diseases clinical trials

In rare diseases research, each observation and data point is particularly precious so that it is important to connect knowledge gathered by different methods and actors. Ideally, data collected directly from patients through mobile technology or wearable biosensors could be readily integrated with data gathered in registries or clinical studies. This would also make it possible to have remote clinical trial visits. Because patients often have to travel across long distances to reach a trial site, and because their condition may make travel challenging, the opportunity to complete some trial visits remotely would be helpful.

Another important barrier can be the lack of a suitable biomarker. Large clinical trials with robust concurrent control cohorts are often not feasible given the small number of patients available. This makes it particularly advantageous to have a laboratory or imaging marker that would help assess efficacy.

### Platform and collective research approaches

Given the thousands of rare diseases in need of research advances and novel therapeutics, it is challenging to envision how we could as a society have sufficient resources to reach all patients. Yet, the scientific opportunities and technologies are increasingly available. Ideally, we would find ways to develop platforms so that some of the infrastructure, data and experience that is common to several products can be shared in the early stages, prior to differentiation during a subsequent competitive phase. Such an approach might also need innovative trial designs such as adaptive designs, master protocols, basket trials, or personalized clinical outcomes assessments [27].

### Leveraging large “precision” medicine and genotyping/phenotyping initiatives

*Several “precision” medicine initiatives have been launched to examine small subsets of patients in terms of their genotypes and phenotypes, including their response to environmental factors and treatments.* The twentieth century saw the introduction of new therapeutics that dramatically improved outcomes for many common and some rare diseases, contributing to an increase in human life expectancy. However, for most drugs we only know that they work “on average”. A more complete understanding of genotypic or other characteristics that improved diagnosis and prediction of drug safety and efficacy would allow for a more tailored, individualized approach. The principles of “precision medicine” are those of rare disease medicine, and each has much to learn from the other. Both depend on effective multi-site networks that allow the study of patient cohorts that are geographically distributed, yet phenotyped, genotyped, and treated under harmonized procedures and data standards. Such networks require innovative ways of accessing and sharing data across institutions, companies, and systems. They require innovative processes that avoid delays and waste due to bureaucratic redundancies, such as duplicative contract negotiations and IRB reviews.

### Public sector-private sector parternships

For rare diseases and for subsets of common diseases alike, government and academic researchers alone can rarely bring new treatments to market and make them available to patients in need. Therefore, it is important that we find effective ways for smooth “hand-offs” between partners, and frameworks for collaborations between patient groups, academics and industry that are transparent and adequately manage any conflicts of interest. The NeuroNEXT initiative which has successfully completed studies on rare diseases such as spinal muscular atrophy features a Cooperative Research and Development Agreement (CRADA) mechanism by which industry can access the NIH-funded NeuroNEXT network infrastructure. Several private-public partnerships were successfully implemented in this manner. However, the different timeline expectations for industry and NIH posed a challenge which was prohibitive to several potential partnerships. The NIH application and peer review process typically requires at least 9 months of lead time. Accommodating this delay was often not feasible for industry partners. In addition to innovations in technology, pre-clinical and clinical development, we need new collaboration frameworks so that all people with rare diseases can ultimately benefit from the fruits of biomedical research.

## Conclusion

Looking ahead, there is great hope for people living with rare diseases. An increasing number of patients are being enrolled in rare diseases clinical trials, and each year a greater number of new rare diseases treatments are approved in the US and internationally.

To ensure that ultimately all rare diseases patients can benefit from the promise of recent scientific advances, we need more efficient and effective models for therapy development that are scalable and sustainable. Such models must accelerate processes and timelines, promote platform approaches, stimulate innovation in manufacturing, regulatory science and trial design, and foster greater synergy among the different stakeholders that are needed to successfully develop new treatments.

## Abbreviations

AHRQ: Agency for Healthcare Research and Quality
CAR-T: Chimeric antigen receptor T cells
CDEs: Common data elements
CPAG: Coalition of Patient Advocacy Groups
CRADA: Cooperative research and development agreement
CRISPR: Clustered regularly interspaced short palindromic repeats
CTSA: Clinical and Translational Science Awards
EHR: Electronic health records
FDA: Food and Drug Administration
GARD: Genetic and Rare Diseases Information Program
HPO: Human Phenotype Ontology
IRB: Institutional Review Board
IRDiRC: International Rare Diseases Research Consortium
NCATS: National Center for Advancing Translational Sciences
NeuroNEXT: Network for Excellence in Neuroscience Clinical Trials
NHGRI: National Human Genome Research Institute
NIH: National Institutes of Health
ORDR: Office of Rare Diseases Research
PCORI: Patient-centered Outcomes Research Initiative
RaDaR: Rare Diseases Registries Program
RDCRN: Rare Diseases Clinical Research Network
RNAi: RNA interference
US: United States
ZNF: Zinc finger
